# Urinary iodine concentration and its associations with thyroid function in pregnant women of Shanghai

**DOI:** 10.3389/fendo.2023.1184747

**Published:** 2023-07-04

**Authors:** Yiming Wu, Jie Yang, Qing Su, Hongxia Gu, Li Qin

**Affiliations:** ^1^ Department of Endocrinology, Chongming Hospital Affiliated to Shanghai University of Medicine and Health Sciences, Shanghai, China; ^2^ Department of Endocrinology, Xinhua Hospital Affiliated to Shanghai Jiaotong University School of Medicine, Shanghai, China

**Keywords:** pregnant women, iodine nutrition, urinary iodine concentration, thyroid hormones, thyroid diseases

## Abstract

**Objective:**

To assess the iodine status and its associations with thyroid function in pregnant women of Shanghai.

**Methods:**

In this cross-sectional study, a total of 562 pregnant women were enrolled from January to December 2021. Both serum thyroid-stimulating hormone (TSH), free triiodothyronine (FT3), free thyroxine (FT4), thyroid peroxidase antibody (TPOAB), thyroglobulin antibody (TGAB), and urinary iodine concentration (UIC) were detected. Participants were divided into four groups based on their UIC values. Correlation analysis was used to investigate the association between UIC and thyroid function-associated parameters.

**Results:**

The median UIC of the pregnant women studied was 158.25µg/L (interquartile range [IQR] 90.15, 245.65µg/L). Among all the subjects, 45.55% had iodine deficiency according to the World Health Organization (WHO) criteria, and 15.65% had thyroid autoimmunity. FT3, FT4, TSH, TPOAB and TGAB levels were not different among different UIC groups (P > 0.05). UIC and TSH were negatively correlated (r=-0.127, p=0.043) in UIC<150 µg/L group. In the group with UIC 250- 499 µg/L, UIC was positively correlated with total T4 (TT4), total T3 (TT3) and TPOAB (r= 0.228, p=0.022, r=0.208, p= 0.039, r=0.190, p=0.042, respectively). A negative correlation between UIC and TPOAB values was observed in TPOAB-positive (+) pregnant women (r=-0.384, p=0.012). The prevalence of isolated hypothyroxinemia in UIC<150 µg/L group was significantly higher than that of other groups(p=0.033). The relationship between the prevalence of thyroid diseases and UIC embodied a U-shaped curve.

**Conclusion:**

Pregnant women on Chongming Island of Shanghai were iodine sufficient during the second trimester, but iodine deficiency was still prevalent. Both low and high gestational iodine status was related to thyroid function and autoimmunity. Optimal iodine nutrition status during gestation was important.

## Introduction

Iodine is an essential element required for the synthesis of thyroid hormones, which play a vital role not only in various metabolic processes throughout life but also in the growth and development of the brain (1). Iodine deficiency is prevalent in pregnant women worldwide due to the increased demand for iodine and the additional renal excretion of iodine during pregnancy. It is worth noting that both inadequate and excessive iodine exposure during pregnancy can affect thyroid function and consequently lead to adverse outcomes for the mother as well as the fetus (2–4).Therefore, iodine nutrition during pregnancy has been recognized as a worldwide public health concern. The World Health Organization (WHO) recommended a daily iodine intake of 250μg for pregnant women (5). About 90% of dietary iodine is excreted in urine and urinary iodine concentration (UIC) is considered a reliable indicator of recent iodine intake. Shanghai is a coastal city where there is an epidemic of iodine deficiency disorders among pregnant women (6, 7). According to a recent epidemiological study in Shanghai, 68.9% of the pregnant women had UICs below 150μg/L, most of which were mild iodine deficiency (8). With a backward economy, poor transport, and low literacy levels, the residents of Chongming Island, the backyard of Shanghai, were lack of proper guidance on iodine intake. We investigated and learned that many locals, regardless of thyroid diseases, consumed non-iodized salt because they believed that iodized salt intake could cause or aggravate thyroid disorders. Meanwhile, community physicians did not conduct iodine nutrition education among the residents. Therefore, few locals were aware of the health effects of low and high iodine intakes. Besides, most country doctors advised people with autoimmune thyroid diseases or thyroid nodules to avoid iodized salt and seafood. As far as we know, no relevant article has reported or analyzed the iodine nutrition of pregnant women on Chongming Island. Therefore, it is necessary to explore the iodine status and its associations with thyroid function in pregnant women to deter iodine-related disorders.

## Subjects and methods

### Subjects

This cross-sectional study enrolled 562 consecutive pregnant women who visited the Chongming Hospital Affiliated to Shanghai University of Medicine and Health Sciences for their antenatal checkup during the second trimester of gestation from January to December 2021. Baseline information including name, age, gestational weeks, gravidity and parity times, and history of diseases was inquired and recorded by a questionnaire. Subjects were recruited if they were 18-40 years old, had at least 5 continuous years of residency in Chongming, and had a singleton pregnancy. Pregnant women with known thyroid or other chronic diseases, multiple pregnancies, and those who had supplemented iodine during pregnancy were excluded from the study. Informed consent was obtained from all participants. The study was approved by the ethics committees of the Chongming Hospital Affiliated to Shanghai University of Medicine and Health Sciences.

### Sample collection and analysis

Venous blood and spot midstream urine samples were collected in the morning from each subject after an overnight fast. Blood and urine samples were sealed and stored at -80°C for the subsequent analysis. Serum thyroid-stimulating hormone (TSH), free thyroxine (FT4), free triiodothyronine (FT3), total T4 (TT4), total T3 (TT3), thyroid peroxidase antibody (TPOAB) and thyroglobulin antibody (TGAB) levels were determined using the chemiluminescent immunoassay (Siemens Healthcare Diagnostics, Germany). UIC was measured by arsenic cerium catalytic spectrophotometry (Chinese Standard WS/T107.1-2016). The iodine status of pregnant women was estimated by the recommended WHO criteria defined as inadequate iodine intake (UIC<150 µg/L), adequate iodine intake (UIC 150-249µg/L), more than adequate iodine intake (UIC 250-499µg/L) and excessive iodine intake (UIC≥500µg/L) (9).

### Diagnostic criteria

Thyroid diseases including Clinical hypothyroidism, subclinical hypothyroidism, clinical hyperthyroidism, subclinical hyperthyroidism, and isolated hypothyroxinemia were diagnosed in line with the Guidelines for the Diagnosis and Management of Thyroid Disease during Pregnancy and the Postpartum (10). The reference ranges of FT4 and TSH for pregnant women in the second trimester were 10.6-17.6pmol/l and 0.05-4.5mU/L, respectively. Clinical hypothyroidism was defined as TSH>4.5mU/L and FT4<10.6pmol/L, subclinical hypothyroidism was defined as TSH>4.5mU/L and FT4 10.6-17.6pmol/L, clinical hyperthyroidism defined as TSH<0.05mU/L and FT4>17.6pmol/L, subclinical hyperthyroidism was defined as TSH<0.05mU/L and FT4 10.6-17.6pmol/L, isolated hypothyroxinemia was defined as TSH 0.05-4.5mU/L and FT4<10.6pmol/L. TPOAB-positive(+) and TGAB-positive (+) were defined as values>34IU/mL and values>13IU/mL, respectively. Subjects with at least one of the two positive thyroid antibodies were defined as TPOAB/TGAB-positive (+) and the others were defined as TPOAB/TGAB-negative (–).

### Statistical analysis

Statistical analysis was performed using SPSS 22.0 software (SPSS, Chicago, IL). The Kolmogorov-Smirnov test was used to detect a normal distribution data. Continuous variables were presented as mean ± standard deviation (SD) or median (interquartile range, IQR). Mann-Whitney’s U-test and the Kruskal-Wallis test were performed for comparisons of UIC and thyroid parameters between groups. Categorical variables were described as frequencies and comparisons among groups used the χ ^2^ or Fisher’s exact test. Spearman’s method was conducted for correlation analysis of UIC and other variables. P < 0.05 was considered statistically significant.

## Results

### Cohort characteristics

**Figure 1:**
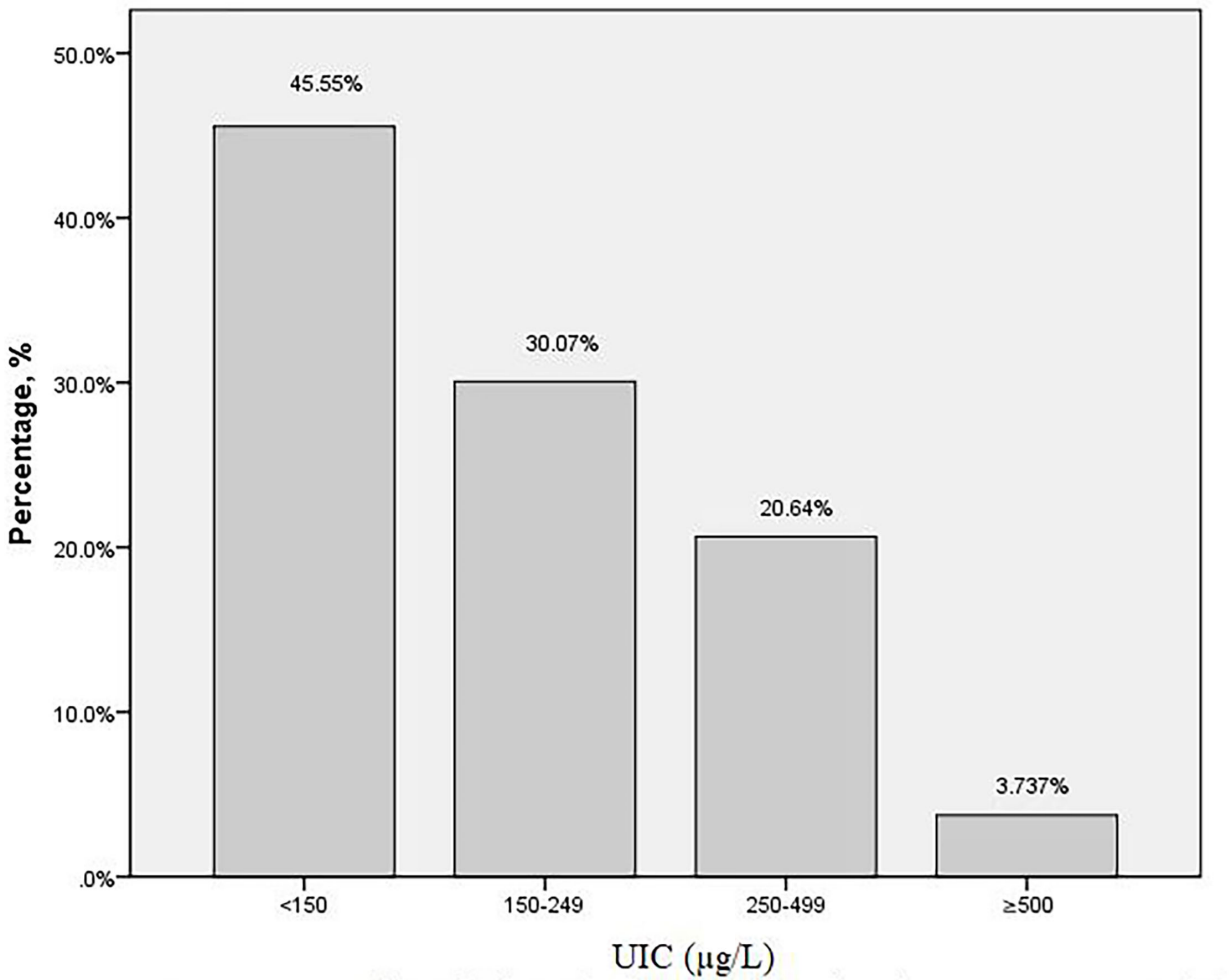
Proportion of urinary iodine concentrations in pregnant women (%).

The baseline characteristics of the subjects were presented in **Table 1**. A total of 562 pregnant women at their second trimester were included in this study. The mean age of the participants was 28.71 ± 4.52 years old. The gestational age at screening was 17.01 ± 1.65 weeks. The overall median (IQR) UIC was 158.25μg/L (90.15, 245.65). Among the 562 subjects, the distribution of UIC < 150μg/L,150–249μg/L,250–499μg/L, and ≥500μg/L was 256 (45.55%),169 (30.07%), 116 (20.64%) and 21 (3.74%), respectively (**Figure 1**). There were 42(7.53%) subjects with TPOAB (+), 51(10.45%) with TGAB (+) and a total of 77 (15.65%) with TPOAB/TGAB (+) status.

**Table 1:** Characteristics, thyroid-related indicators and UIC of pregnant women (n=562).

Variable	Values
Age, years	28.71 ± 4.52
Gestational age, weeks	17.01 ± 1.65
Parity times	2 (1–3)
Delivery times	0 (0–1)
BMI, kg/m2	22.27 ± 4.60
TSH, mU/L	1.44 (1.00,2.04)
FT4, pmol/L	14.58 (12.96,16.30)
FT3, pmol/L	4.46 (4.19,4.71)
TT4, pmol/L	119.80 (96.15,141.18)
TT3, pmol/L	2.01 (1.49,2.36)
TPOAB (+), n (%)	42 (7.53%)
TGAB (+), n (%)	51 (10.45%)
TPOAB/TGAB (+), n (%)	77 (15.65%)
UIC, μg/L	158.25 (90.15, 245.65)

Values are expressed as mean± standard deviation (SD) or median (IQR).

BMI, body mass index; UIC, urinary iodine concentration; FT3, free triiodothyronine; FT4, free thyroid hormone; TSH, thyroid-stimulating hormone; TT3, total triiodothyronine; TT4, total thyroxine; TPOAB, thyroid peroxidase antibody; TGAB, thyroglobulin antibody; TPOAB (+), TPOAB-positive; TGAB (+), TGAB-positive.

### Serum thyroid hormones and antibodies values of pregnant women stratified by UIC

Serum levels of TSH, FT4, FT3, TGAB and TPOAB of the subjects stratified by UIC were listed in **Table 2**. There was no significant difference of TSH, FT4, FT3 levels among different UIC groups. TGAB and TPOAB values were higher in UIC <150μg/L and ≥500μg/L group than that in UIC 150–249μg/L group, but the difference did not reach statistical significance (P>0.05, **Table 2**).

**Table 2:** Serum thyroid hormones and antibodies values in different UIC groups.

UIC (µg/L)	TSH (mU/L)	FT3 (pmol/L)	FT4 (pmol/L)	TPOAB (IU/mL)	TGAB (IU/mL)
<150	1.46(0.98,2.07)	4.45(4.20,4.70)	14.80 (12.86,16.37)	14.98(10.32,21.22)	7.10(5.85,9.52)
150–249	1.51(1.11,2.17)	4.43(4.15,4.72)	14.62 (13.08,16.10)	14.97(10.50,20.76)	6.77(5.64,8.08)
250–499	1.40(0.94,1.96)	4.55(4.19,4.91)	14.53 (12.98,16.58)	14.89(10.49,19.73)	7.00(5.87,8.36)
≥500	1.46(1.17,1.70)	4.41(4.15,4.71)	13.88 (13.08,17.28)	15.11(10.88,21.67)	7.31(5.97,7.98)
P value	0.495	0.442	0.995	0.938	0.371

Data are reported as median (IQR). Significance between subgroups using Kruskal-Wallis H-test, P value significance set at P < 0.05.

### UIC and thyroid function of subjects with different types and titers of thyroid antibodies

**Table 3** descripted UIC and thyroid hormones in terms of TPOAB/TGAB positivity. The median (IQR) UIC of subjects with TPOAB (+) and TPOAB (-) was 125.40μg/L (77.60,190.78) and 161.40μg/L (91.80,251.20) respectively, the difference was significant (p=0.027). The median (IQR) UIC in TGAB (+) group was 125.00μg/L (72.70,212.20), which was significantly lower than that of subjects with TGAB (-) (p=0.040). In TPOAB/TGAB (+) subjects, the UIC value was 125.00μg/L (73.60,202.10), which was also lower compared with double antibody (-) subjects (p=0.018). While, we didn’t find significant difference in term of TSH, FT4, FT3 levels among subjects with and without TPOAB/TGAB (+) (P>0.05, **Table 3**).

**Table 3:** UIC and thyroid function of pregnant women stratified by TPOAB/TGAB positivity.

Groups	TSH (mU/L)	FT3 (pmol/L)	FT4 (pmol/L)	UIC (µg/L)
TPOAB (+)	1.59(1.02,2.03)	4.39(4.12,4.71)	14.12 (12.91,16.20)	125.40(77.60,190.78)
TPOAB (-)	1.44(1.00,2.04)	4.47(4.19,4.72)	14.64 (12.97,16.34)	161.40(91.80,251.20)
P value	0.428	0.610	0.597	0.027
TGAB (+)	1.46(1.10,2.01)	4.41(4.13,4.83)	14.73(13.22,17.96)	125.00(72.70,212.20)
TGAB (-)	1.45(1.00,2.07)	4.48(4.19,4.72)	14.71(12.97,16.32)	162.00 (94.400,251.80)
P value	0.954	0.802	0.257	0.040
TPOAB/TGAB (+)	1.49(1.05,2.00)	4.40(4.14,4.71)	14.42(12.92,16.35)	125.00(73.60,202.10)
TPOAB and TGAB (-)	1.45(1.00,2.08)	4.48(4.19,4.72)	14.76(12.98,16.37)	163.30(95.10,256.30)
P value	0.900	0.596	0.863	0.018

Data are reported as median (IQR). Significance between subgroups using Mann-Whitney test. P value significance set at P < 0.05.

### Relationship between UIC and thyroid-related indicators

**Figure 2:**
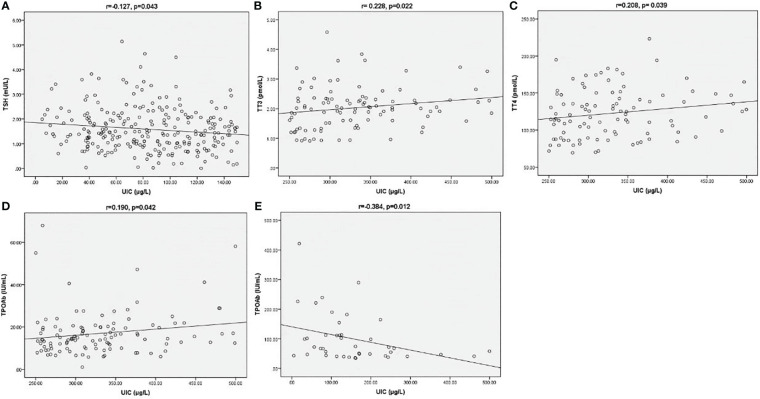
Correction between UIC and TSH **(A)** in UIC<150 µg/L group. Correction between UIC and TT3 **(B)**, TT4 **(C)** and TPOAB **(D)** in UIC 250-499 µg/L group. Correction between UIC and TPOAB **(E)** in TPOAB (+) group. The Spearman correlation coefficient (r) and the level of significance (p) were marked above the respective graph.

Correlations among UIC and thyroid-related indicators were depicted on scatter plots in **Figure 2**. In UIC<150 µg/L group, there was a negative correction between UIC and TSH (r=-0.127, p=0.043, **Figure 2A**). In the subjects with UIC 250- 499 µg/L, UIC was positively correlated with TT3 and TT4 and TPOAB values (r= 0.228, p=0.022, r=0.208, p= 0.039, r=0.190, p=0.042, respectively, **Figures 2B-D**). There was no significant correlation between UIC and T3, T4, FT3, FT4, TSH in groups with UIC 150- 249 µg/L and UIC≥500 µg/L (P>0.05). In TPOAB (+) subgroup, we found a negative correlation between UIC and TPOAB values (r=-0.384, p=0.012, **Figure 2E**). In TGAB (+) group, no significant correlation was observed between UIC and other parameters.

### Relationship between UIC and thyroid diseases

**Table 4** depicted the prevalence of thyroid diseases in pregnant women stratified according to UIC range. We found significant difference in the prevalence of isolated hypothyroxinemia between the four groups (p=0.033), the highest prevalence of isolated hypothyroxinemia (7.4%) was observed in UIC<150 µg/L group. While, there was no significant difference in the prevalence of other thyroid diseases among groups with different UIC (p>0.05). A U-shaped tendency for the prevalence of thyroid diseases as UIC increased and decreased could also be observed in **Table 4**. The overall prevalence of thyroid diseases in other groups was higher than that of in UIC 150-249 µg/L group, this difference, however, did not reach statistical significance among groups (p>0.05, **Table 4**).

**Table 4:** Prevalence of thyroid diseases in pregnant women with different UIC, n (%).

UIC groups	<150 (µg/L)	150 – 249 (µg/L)	250 – 499 (µg/L)	≥500 (µg/L)	P value
Thyroid diseases					
Clinical hypothyroidism	0 (0.00)	1 (0.59)	1 (0.86)	0 (0.00)	0.330
Subclinical hypothyroidism	3 (1.17)	5 (2.96)	4 (3.45)	1 (4.76)	0.245
Clinical hyperthyroidism	6 (2.34)	3 (1.78)	4 (3.45)	0 (0.00)	0.797
Subclinical hyperthyroidism	14 (5.46)	5 (2.96)	2 (1.72)	2 (9.52)	0.136
Isolated hypothyroxinemia	19 (7.42)	4 (2.37)	2 (1.72)	1 (4.76)	0.033
Total	42 (16.41)	18 (10.65)	13 (11.21)	4 (19.05)	0.258

Data were expressed as n (%). Comparisons between groups were performed using a Chi-square test or Fisher’s exact test with respect to the prevalence of thyroid diseases. P value significance set at P < 0.05.

## Discussion

To our knowledge, the current study was the first to investigate iodine nutrition and its relationships with thyroid function and autoimmunity of pregnant women on Chongming Island of Shanghai.

Our study showed that the median UIC (IQR) of pregnant women was 158.25μg/L (90.15, 245.65), which was at the lower limit of the iodine adequate range. Although the result showed that iodine nutrition was sufficient, we looked further into the UIC stratification in all participants. We discovered that nearly half (45.55%) of the subjects were indicative of iodine deficiency in their second trimester of gestation according to the WHO stipulated UIC cut-off of 150μg/L. Our result was in line with previous data derived from a larger sample size of 2400 pregnant women in Shanghai, which showed that the proportion of iodine deficiency was 48% (11). Apparently, the phenomenon of iodine deficiency was widespread among pregnant women in Shanghai and most of them were in a situation of mild iodine deficiency (12). Pregnant women were vulnerable to iodine deficiency. Despite the increased requirements and excretions of iodine during pregnancy, another possible reason was that pregnant women consumed non-iodized salt and reduced their seafood intake. Previous monitoring results presented that the median UIC of pregnant women was 126.52μg/L in 2015, significantly lower than that of 2011(139.77μg/L) in Shanghai. The drop might be attributed to the declining coverage rate of iodized salt, which decreased by 17.7% from 2011 to 2015 (13). Additionally, in Shanghai, the median UIC of pregnant women in 2014 was 20% and 9.5% lower than that in 1999 and 2011, respectively. There was a downward trend in terms of iodized salt concentration (14). Notably, pregnant women in Shanghai who consumed non-iodized salt had higher educational status, economic level, previous thyroid disease rate, first pregnancy rate, and elderly maternal rate, meanwhile, they had little or no intake of iodine-rich foods (15). Given these details, the need for ongoing monitoring of gestational iodine status was of great importance.

The relationship between gestational iodine status and thyroid function has been the focus of clinical research in recent years. In this study, we found that thyroid function did not differ substantially according to the UIC range. A possible explanation was that the majority of iodine deficiency subjects were in a mild degree in our study, which might not affect thyroid function markedly because of the activated compensatory mechanism of the thyroid glands to maintain the synthesis of thyroid hormones (16). On the other hand, an excessive iodine load inhibited thyroid hormone formation and secretion, a process known as the Wolff-Chaikoff effect (17). Early studies explored the associations between iodine and thyroid function during pregnancy in different iodine nutrition regions, the results of which were conflicting. One study conducted in an iodine-sufficient region in Japan observed significant positive correlations between UIC and TSH whereas negative correlations between UIC and FT4 (18). Evidence from a recent paper in Chongqing pointed out that TSH and UIC were positively correlated, while no relationship was observed between UIC and serum FT3 and FT4 levels (19). Another study reported no association between UIC and any parameters of thyroid function in early pregnant women (20). In agreement with this result, Blumenthal found no association between UIC and thyroid function in pregnant women in a mild-iodine-deficient region (21). In this present study, a negative correlation between UIC and TSH was observed in the UIC<150 µg/L group. Due to the body’s feedback metabolism, iodine deficiency could stimulate the pituitary to produce more TSH to maintain thyroid hormone synthesis. Meanwhile, in the group with UIC 250-499µg/L, UIC values were positively correlated with TT3 and TT4. It indicated that increased exposure to iodine could promote the synthesis of thyroid hormones. While in UIC≥500 µg/L group, the correction was not remarkable. A possible reason was that excessive iodine intake would inhibit thyroid hormone synthesis to deter thyrotoxicosis. The cause-and-effect relationship between the two was not well investigated, therefore, further studies should be performed.

Thyroid autoimmunity was common among women. TPOAB and TGAB were regarded as the primary pathogenic thyroid antibodies in autoimmune thyroiditis (AIT), including Hashimoto’s thyroiditis (HT). Epidemiological and clinical evidence on the link between iodine and thyroid autoimmunity in pregnant women was inconsistent. The study by Chen et al. found low UIC was an independent risk factor for positive TGAB (22). A cross-sectional study including 7073 pregnant women from an iodine-sufficient region of China also discovered that iodine deficiency was associated with higher risks of TPOAB positivity and TGAB positivity (23). Consistently, in an urban Chinese population, low UIC was reported to be a risk factor for AITD, especially for women (24). Conversely, Rayman et al. drew a conclusion that exposure to excess iodine intake induced thyroid autoimmunity, and the appropriate iodine status was crucial to thyroid health (25). Moreover, one study performed in a large sample of Shanghai found a significantly higher risk of TPOAB/TGAB/TRAB (+) status in pregnant women with low and excessive iodine intakes (15). A similar trend was reported by Shi et al. who also discovered that the prevalence of TPOAB positivity and TGAB positivity presented a U-shaped curve, ranging from mild iodine deficiency to iodine excess (26). Our study assessed the UIC values of pregnant women with and without TPOAB/TGAB (+). It should be noted that the median UICs of the subjects with single antibody positive or double positive were indicative of iodine deficiency and significantly lower than that of antibody negative- subjects, who were iodine sufficient based on the WHO criteria. A recent study conducted in Shanghai showed a result similar to our study. Furthermore, the literature revealed that subjects with thyroid antibodies (+) were more likely to consume non-iodized salt, less iodine-rich foods, and iodine-rich nutritional supplements. Thus, their iodine intake was lower, contributing to iodine deficiency. On the other hand, those with iodine deficiency were reported to have an increased risk of thyroid autoantibodies positivity (15). In light of these findings, we supposed that perhaps strict restriction of dietary iodine intake was inappropriate for pregnant women with AIT. Both iodine deficiency and excess were reported to impact thyroid autoimmunity adversely (26, 27). In our study, UIC was positively correlated with TPOAB value in the UIC 250-499µg/L group, indicating that more than adequate iodine intake had relevance to higher TPOAB titer. We speculated that since there were few women with excessive iodine, no significant association of UIC with thyroid antibodies was identified in the UIC≥500 µg/L group. On the other hand, a negative correlation between UIC and TPOAB values could be observed in TPOAB (+) subjects who were iodine deficiency. Thus, we inferred that there was probably a connection between iodine deficiency and aggravated thyroid autoimmunity. The mechanism of iodine on thyroid autoimmunity was uncertain. It was reported that iodine deficiency might influence thyroid autoimmunity through inflammatory responses induced by oxidative stress, and highly-iodinated thyroglobulin caused by excessive iodine intake was more immunogenic, accelerating the thyroid autoimmune process (25, 28, 29).

As previously reported, iodine deficiency was the most common cause of isolated hypothyroxinemia (30). In our work, the prevalence (7.4%) of isolated hypothyroxinemia was significantly higher in the UIC<150 (µg/L) group compared to other UIC groups. In contrast, one study including 7190 pregnant Shanghai women failed to observe a similar result in the UIC<100 ug/L group. Evidence suggested that the prevalence of thyroid diseases increased in pregnant women with iodine deficiency and iodine excess (19, 31). Similarly, a U-shaped curve for the prevalence of thyroid diseases as UIC increased and decreased could also be observed in this current study, and the prevalence was low when the UIC was within the normal range. The mechanism of iodine on thyroid dysfunction was unclear. Further intensive studies are needed.

Our study had several limitations. First, we collected spot urine samples instead of 24h urine data to assess the iodine nutrition of pregnant women. Second, this cross-sectional study could not establish the causal relationship between maternal thyroid abnormality and iodine status. Finally, we failed to observe iodine nutrition at different periods throughout pregnancy and after delivery.

## Conclusion

In summary, iodine deficiency was prevalent in pregnant women living on Chongming Island, Shanghai. Both low and high gestational iodine status might be associated with thyroid function and autoimmunity. Therefore, it is important and necessary to monitor iodine nutrition status to provide reasonable guidance on iodine intake during pregnancy.

## Data availability statement

The original contributions presented in the study are included in the article/supplementary materials. Further inquiries can be directed to the corresponding authors.

## Ethics statement

The studies involving human participants were reviewed and approved by the ethics committees of the Chongming Hospital Affiliated to Shanghai University of Medicine and Health Sciences. The patients/participants provided their written informed consent to participate in this study.

## Author contributions

LQ and HG defined the research theme. YW performed experiments, collected and analyzed the data and wrote the paper. JY and QS revised the article for important intellectual content. All authors read and approved the final manuscript.
